# Animal models for investigating post‐stroke cognitive impairment: A neurovascular trafficking perspective

**DOI:** 10.1002/ame2.70315

**Published:** 2026-10-18

**Authors:** Pratchaya Kaewkaen

**Affiliations:** ^1^ Animal Cognitive Neuroscience Laboratory (ACoN) Burapha University Chonburi Thailand; ^2^ Department of Research and Applied Psychology, Faculty of Education Burapha University Chonburi Thailand

**Keywords:** animal models, blood–brain barrier, chronic cerebral hypoperfusion, endolysosomal trafficking, extracellular vesicles, ischemic stroke, neurovascular unit, post‐stroke cognitive impairment, vascular cognitive impairment, white matter injury

## Abstract

Post‐stroke cognitive impairment (PSCI) is a major cause of long‐term disability after ischemic stroke, yet its mechanisms remain incompletely explained by infarct volume, lesion location, or conventional vascular risk factors alone. Animal models are indispensable for clarifying how ischemic injury evolves into delayed cognitive dysfunction and for determining which mechanisms are experimentally tractable, reproducible, and translationally relevant. This review examines PSCI from a neurovascular trafficking perspective, focusing on how laboratory animal models can be used to investigate secretory pathway stress, endolysosomal dysfunction, autophagy‐lysosomal flux, extracellular vesicle signaling, blood–brain barrier disruption, glial reactivity, myelin debris clearance, white matter injury, and cognition‐relevant behavioral phenotypes. Rodent models of transient or permanent middle cerebral artery occlusion, photothrombotic stroke, embolic stroke, chronic cerebral hypoperfusion, bilateral carotid artery stenosis, and subcortical white‐matter injury each reproduce selected components of the human PSCI spectrum. No single model fully captures the temporal, vascular, cellular, and cognitive complexity of human PSCI; therefore, model selection should be driven by the mechanistic question, disease phase, vascular pathology, cellular compartment, cognitive domain, and welfare burden under investigation. The central argument is not that neurovascular trafficking failure is already a proven causal pathway, but that well‐designed animal studies can test, refine, or falsify this hypothesis. Future studies should integrate model validity, translational rigor, and welfare refinement through longitudinal cognitive testing, multimodal imaging, cell‐specific molecular profiling, extracellular vesicle phenotyping, intervention‐based validation, randomization, blinding, sample‐size justification, humane endpoints, perioperative care, and transparent reporting.

## Graphical abstract

Animal models enable mechanistic testing of how ischemic injury disrupts neurovascular trafficking and drives cognitive vulnerability after stroke. Complementary models capture distinct vascular, cellular, white matter, and cognitive features of PSCI.

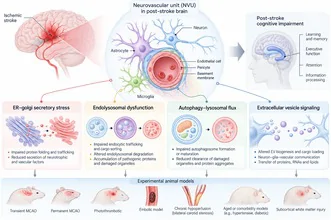

## 1 INTRODUCTION: WHY ANIMAL MODELS ARE NEEDED TO STUDY POST‐STROKE COGNITIVE IMPAIRMENT

Post‐stroke cognitive impairment (PSCI) represents a prevalent and clinically significant sequela of both ischemic and hemorrhagic stroke. Human cohort studies indicate that delayed cognitive decline is influenced by lesion characteristics, vascular risk profiles, pre‐existing cerebral vulnerability, white matter burden, mood disorders, educational attainment, cognitive reserve, and systemic comorbidities.^1^, ^2^, ^3^, ^4^, ^5^, ^6^ However, these variables do not fully account for the substantial inter‐individual variability observed in recovery trajectories and long‐term cognitive outcomes. Consequently, a critical mechanistic gap persists between the initial ischemic insult and the subsequent emergence of deficits in memory, attention, executive function, processing speed, and large‐scale brain network integrity.

The neurovascular unit (NVU) provides a biologically integrated framework for addressing this gap. It comprises endothelial cells, pericytes, astrocytic endfeet, microglia, neurons, oligodendrocyte lineage cells, the basement membrane, and extracellular matrix components. Together, these components form a dynamic and interdependent system. Its coordinated functions are essential for maintaining blood–brain barrier (BBB) integrity, regulating neurovascular coupling, facilitating metabolic exchange, supporting immune surveillance, preserving synaptic function, and enabling tissue repair processes.^7^, ^8^ Within this framework, secretory‐endolysosomal dysfunction is proposed as a hypothesis‐generating axis. This axis may help explain the progression from ischemia‐induced NVU stress to delayed cognitive vulnerability. However, direct causal evidence remains limited.

Animal models are indispensable for investigating these mechanistic questions. They permit temporal and tissue‐level analyses, cell‐type‐specific manipulation, and controlled interventions that are difficult or impossible to perform in human stroke populations. Rodent models, in particular, reproduce key features of PSCI, including focal ischemia, chronic cerebral hypoperfusion, white matter injury, neuroinflammation, hippocampal dysfunction, BBB disruption, and associated cognitive deficits.^9^, ^10^, ^11^, ^12^, ^13^, ^14^, ^15^, ^16^, ^17^, ^18^, ^19^, ^20^, ^21^, ^22^, ^23^, ^24^ Importantly, these models permit direct investigation of subcellular processes implicated in NVU dysfunction, such as endoplasmic reticulum stress, Golgi apparatus disruption, endolysosomal trafficking, autophagy–lysosomal flux, extracellular vesicle (EV) dynamics, and myelin debris clearance.^25^, ^26^, ^27^, ^28^, ^29^, ^30^, ^31^, ^32^, ^33^, ^34^, ^35^, ^36^, ^37^ Nevertheless, animal models do not represent simplified replicas of human PSCI. Their translational value depends on careful alignment between the chosen model and the specific research question, the use of cognitively relevant outcome measures, appropriate control of sensorimotor confounds, and adherence to methodological rigor, including randomization, blinding, sample size justification, welfare considerations, and transparent reporting standards.^38^, ^39^, ^40^, ^41^ This model‐selection approach is consistent with prior reviews in animal models and experimental medicine emphasizing that stroke is pathophysiologically heterogeneous and that no single experimental model reproduces all aspects of human stroke.^42^, ^43^

Accordingly, this review asks: which animal models are best suited to investigate whether secretory‐endolysosomal dysfunction within the ischemic NVU contributes to delayed cognitive impairment? The objective is to refine the conceptual framework into an animal‐model‐centered research roadmap that is organized around three priorities: model validity, translational rigor, and welfare refinement. These priorities help ensure that mechanistic hypotheses are tested in models that match the intended disease feature, use robust experimental design and cognition‐relevant endpoints, and apply refinement strategies that minimize animal burden without compromising scientific interpretation.

## 2 SCOPE AND EVIDENTIARY APPROACH

This article is a hypothesis‐generating narrative review focused on animal models of ischemic stroke, vascular cognitive impairment, and PSCI. It is not presented as a systematic review or meta‐analysis. PubMed‐indexed literature was prioritized during revision, with emphasis on studies and reviews concerning rodent stroke models, chronic cerebral hypoperfusion, vascular cognitive impairment, BBB disruption, NVU dysfunction, endolysosomal trafficking, autophagy‐lysosomal signaling, EV biology, white matter injury, cognitive testing, animal‐model validity, translational rigor, and laboratory animal welfare.

The synthesis uses three interpretive categories. First, established observations refer to mechanisms supported by direct experimental or clinical evidence, such as ischemia‐induced ER stress, BBB disruption, autophagy‐lysosomal disturbance, EV release, and white matter injury.^4^, ^5^, ^6^, ^7^, ^8^, ^25^, ^26^, ^27^, ^28^, ^29^, ^30^, ^31^, ^32^, ^33^, ^34^, ^35^, ^36^, ^37^, ^44^, ^45^, ^46^, ^47^, ^48^, ^49^ Second, model‐supported inferences refer to relationships that have been observed in animals but require validation across laboratories, species, sex, age, comorbidities, and behavioral domains, such as chronic cognitive impairment after transient MCAO, photothrombotic cortical stroke, or BCAS.^9^, ^10^, ^11^, ^12^, ^13^, ^14^, ^15^, ^16^, ^17^, ^18^, ^19^, ^20^, ^21^, ^22^, ^23^, ^24^ Third, hypothesis‐level propositions refer to claims that still require direct causal testing, such as whether secretory‐endolysosomal dysfunction independently predicts PSCI beyond lesion volume, motor impairment, vascular risk, and baseline brain vulnerability. This distinction is important because the central pathway proposed here is not yet a proven linear sequence. Rather, secretory‐endolysosomal dysfunction is presented as an experimentally testable axis that may help organize animal studies of post‐ischemic NVU failure and delayed cognitive decline.

## 3 MODEL VALIDITY, TRANSLATIONAL RIGOR, AND WELFARE REFINEMENT IN PSCI ANIMAL MODELS

In animal‐model research, model validity, translational rigor, and welfare refinement should be treated as linked design principles rather than separate reporting items. Model validity asks whether the selected animal model reproduces the disease component needed to answer the mechanistic question. Translational rigor asks whether the study design, outcome measures, timing, and statistical safeguards are strong enough to support cautious inference toward human PSCI. Welfare refinement asks whether animal burden is minimized through appropriate perioperative care, monitoring, humane endpoints, analgesia when compatible with the scientific question, and reduction of unnecessary procedures.

For an animal model to be useful in PSCI research, its validity should be defined in relation to the question being asked rather than assumed from the presence of an ischemic lesion alone. Face validity refers to whether the model reproduces observable features of the human condition, such as delayed cognitive impairment, white matter injury, BBB dysfunction, or chronic glial reactivity. Construct validity refers to whether the biological process used to generate the model reflects a relevant disease mechanism, such as focal ischemia, reperfusion injury, chronic hypoperfusion, or subcortical vascular injury. Predictive validity refers to whether interventions that improve animal outcomes have plausible relevance to human disease mechanisms and endpoints.^9^, ^18^, ^19^, ^20^, ^21^

This distinction is particularly important for PSCI because models that are strong for infarct biology may be weak for delayed cognition, whereas models that reproduce white matter vulnerability or chronic hypoperfusion may be less suitable for acute reperfusion biology. For example, transient MCAO is useful for studying acute‐to‐chronic trajectories after focal ischemia, whereas BCAS and related hypoperfusion models are more appropriate for examining vascular cognitive impairment, white matter pathology, and prolonged NVU stress.^10^, ^11^, ^12^, ^13^, ^14^, ^19^, ^20^, ^21^, ^22^, ^23^, ^24^ Photothrombotic models can be valuable for reproducible lesion placement and cross‐species cognitive phenotyping, but they have limitations for modeling thromboembolic and reperfusion processes^15^, ^16^ (Figure 1). The comparative strengths, limitations, mechanistic applications, and recommended assessment timeframes of the principal PSCI animal models are summarized in Table 1.

**FIGURE 1:**
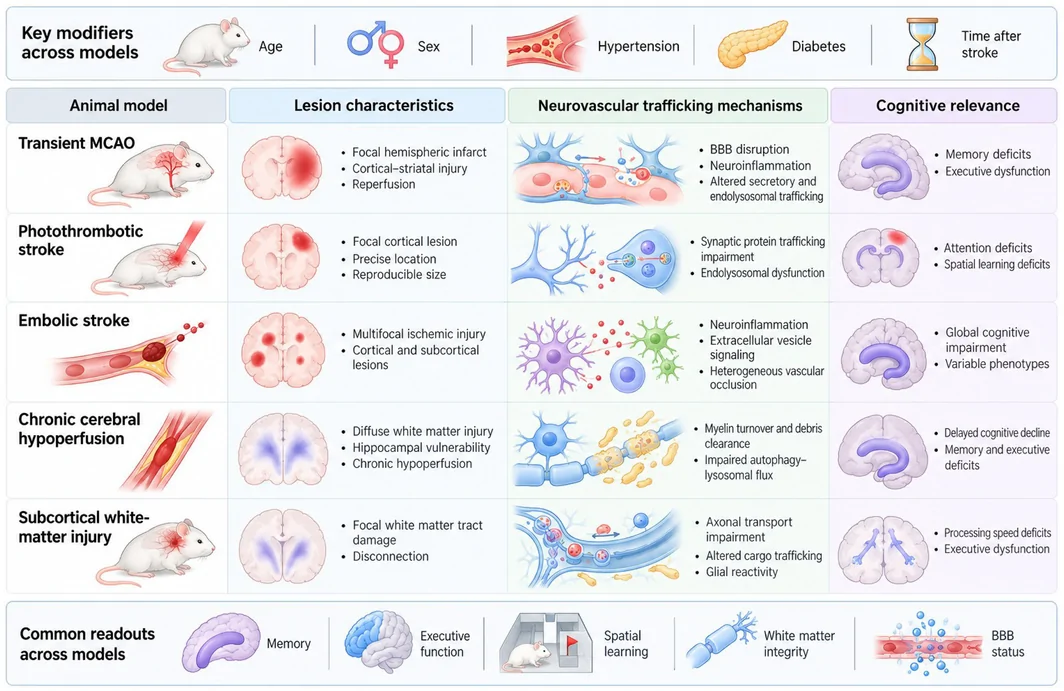
Comparative overview of animal models and pathological features relevant to post‐stroke cognitive impairment.

**TABLE 1:** Alignment between animal models and mechanistic questions for the proposed NVU framework.

Animal model/paradigm	Disease feature modeled	Strength for PSCI research	Key limitations	Best use in this framework	Recommended timeframe for cognitive and molecular readouts
Transient MCAO/reperfusion	Focal ischemia with reperfusion injury, peri‐infarct stress, delayed remote effects	Useful for testing acute‐to‐chronic trajectories, ER stress, BBB disruption, inflammation, and later cognition^10^, ^11^	Sensorimotor deficits may confound cognitive tasks; infarct variability; often young healthy animals	Test whether early trafficking, lysosomal, and BBB stress predicts delayed cognitive dysfunction across the acute‐to‐chronic post‐stroke trajectory	*Molecular*: acute (hours–3 days), subacute (7–14 days), and chronic (≥28 days). *Cognition*: preferably after substantial sensorimotor recovery, typically from 2 to 4 weeks onward, with chronic follow‐up
Permanent MCAO or distal MCAO	Sustained focal ischemia; cortical and striatal injury depending on method	Useful for stable infarct models and chronic tissue remodeling	May overrepresent severe tissue loss relative to subtle PSCI; cognitive domains depend on lesion location	Compare lesion‐dependent and lesion‐independent NVU secretory–endolysosomal changes during chronic tissue remodeling and cognitive decline	*Molecular*: acute to subacute phases for infarct‐associated signaling and chronic phases for remodeling. *Cognition*: subacute‐to‐chronic testing after stabilization of neurological deficits
Photothrombotic cortical stroke	Highly reproducible cortical lesions; remote hippocampal and network effects	Well suited for touchscreen and domain‐specific cognitive testing^15^, ^16^	Limited penumbra and vascular reperfusion biology; lesion may be artificial compared with thromboembolic stroke	Well suited for dissecting remote hippocampal and network‐level effects with relatively limited sensorimotor confounding, particularly in touchscreen‐based cognitive tasks	*Molecular*: acute peri‐infarct responses and subacute/chronic remote hippocampal or network changes. *Cognition*: approximately 2–8 weeks, depending on lesion location and task
Embolic/thromboembolic stroke	Closer to clinical occlusion‐reperfusion and clot‐related pathophysiology	Translational relevance for reperfusion and comorbidity studies^14^	Greater variability; more demanding technical control	Evaluate EV release, vascular inflammation, BBB repair, and secretory ‐endolysosomal responses in a clinically relevant thromboembolic and reperfusion context	*Molecular*: early hours–days for vascular, reperfusion, EV, and inflammatory responses; later weeks for repair. *Cognition*: after stabilization of acute neurological deficits, with chronic follow‐up
BCAS/chronic cerebral hypoperfusion	Gradual hypoperfusion, white matter injury, vascular cognitive impairment	Strong relevance to VCI, white matter injury, and executive‐memory deficits^19^, ^20^, ^23^	Not an acute focal stroke model; time course differs from post‐stroke decline	Test whether prolonged NVU trafficking and lysosomal stress contribute to white matter vulnerability, network dysfunction, and progressive cognitive impairment	*Molecular*: serial subacute and chronic assessments during progressive hypoperfusion and white‐matter injury. *Cognition*: repeated testing over weeks to months
Subcortical/white matter infarct models	Lacunar/subcortical injury, network disconnection, dementia‐like phenotype	Highly relevant to white matter degeneration and cognitive decline^21^	Surgical complexity; limited availability across laboratories	Link impaired lysosomal debris clearance and oligodendroglial injury to disrupted white matter connectivity and cognition‐relevant network dysfunction	*Molecular*: subacute and chronic assessment of myelin injury, glial clearance, and network remodeling. *Cognition*: delayed testing during established white‐matter dysfunction
Aged, hypertensive, diabetic, or mixed‐pathology models	Clinical modifiers of PSCI risk	Improves external validity and translational relevance^13^, ^14^	Higher variability, mortality, cost, and welfare burden	Determine whether aging and vascular comorbidities amplify secretory–endolysosomal vulnerability and modify the relationship between NVU dysfunction and cognitive outcomes	*Baseline*: pre‐ischemic molecular and cognitive characterization is strongly recommended. *Post‐stroke*: acute, subacute, and chronic longitudinal assessments to distinguish pre‐existing vulnerability from stroke‐induced change

Translational alignment therefore requires matching the animal model, behavioral task, molecular readout, and time point to the intended human PSCI feature. A study focused on secretory pathway stress should include temporal ER‐Golgi markers and downstream tissue outcomes. A study focused on endolysosomal clearance should measure flux rather than relying solely on static marker expression. A study focused on EV‐mediated communication should include cell‐of‐origin assignment, cargo characterization, and functional uptake assays. Across all designs, cognitive testing should be interpreted together with motor, visual, motivational, and anxiety‐related controls.

Methodological validity is equally important and should be considered a prerequisite for translational rigor. Randomization, allocation concealment, blinded outcome assessment, sample‐size justification, prespecified exclusion criteria, transparent mortality reporting, and cross‐domain behavioral assessment are not merely technical details; they determine whether an apparent mechanistic association can be interpreted beyond injury severity, lesion variability, or behavioral confounding.^38^, ^39^, ^40^, ^41^, ^42^, ^43^ Welfare refinement should be embedded in the same design logic by specifying monitoring frequency, hydration and nutritional support, body‐temperature control, perioperative care, humane endpoints, and analgesic strategies that reduce distress while preserving valid neurological and cognitive readouts.

## 4 ANIMAL MODELS AS TOOLS TO TEST THE SECRETORY‐ENDOLYSOSOMAL NVU HYPOTHESIS

A useful animal model should reproduce the component of human disease that is central to the research question. For PSCI, the relevant components include focal or diffuse vascular injury, delayed cognitive impairment, BBB dysfunction, glial reactivity, synaptic or hippocampal remodeling, white matter vulnerability, and network‐level disconnection. Because these features do not emerge uniformly across all models, model selection should be hypothesis‐driven rather than convenience‐driven.

Transient MCAO remains one of the most widely used models for focal ischemia and reperfusion injury. It is useful for linking acute cellular stress to subacute and chronic outcomes because tissue can be sampled at defined time points after ischemia. Experimental work has shown that transient focal ischemia can induce long‐term cognitive deficits in rats and mice, and animal studies have linked chronic PSCI‐like phenotypes to inflammasome activation, hippocampal neurogenesis, hypertension, diabetes, and remote neurovascular remodeling.^10^, ^11^, ^12^, ^13^, ^14^ These models are appropriate for testing whether early ER‐Golgi stress, endolysosomal trafficking interruption, lysosomal overload, or EV cargo changes precede later cognitive impairment. Recent AMEM work on Mongolian gerbil ischemia–reperfusion modeling further illustrates how standardized model‐development and evaluation systems can improve reproducibility and model‐specific interpretation in cerebral ischemia research.^50^

Photothrombotic stroke models are especially useful when reproducibility and long‐term behavioral testing are priorities. Although photothrombosis does not fully reproduce the thromboembolic or reperfusion biology of human stroke, it can produce stable cortical lesions and delayed cognitive impairment. Touchscreen‐based studies have shown that mice and human stroke survivors can be compared using cognition‐relevant tasks, strengthening the translational value of carefully selected behavioral paradigms.^15^, ^16^ These models are therefore useful for studying remote hippocampal dysfunction, secondary degeneration, and the relationship between peri‐infarct cellular stress and cognition‐relevant networks.

Chronic cerebral hypoperfusion models are highly relevant to vascular cognitive impairment and white matter vulnerability. Rat 2‐vessel occlusion, mouse bilateral common carotid artery stenosis (BCAS), asymmetric carotid surgery, and related hypoperfusion models produce varying degrees of cerebral blood‐flow reduction, white matter injury, glial activation, and cognitive impairment.^18^, ^19^, ^20^, ^21^, ^22^, ^23^, ^24^ The BCAS model is particularly valuable for studying gradual white matter damage, executive and memory deficits, and the interaction between BBB dysfunction, microglial activation, oligodendroglial stress, and myelin debris clearance.^19^, ^20^, ^23^ These models can test whether chronic NVU trafficking stress produces a prolonged failure of communication and clearance, rather than only an acute response to infarction.

Subcortical infarct and white matter injury models are important because human PSCI is often shaped by network disconnection rather than by cortical infarct volume alone. Models of subcortical infarcts with dementia‐like phenotypes allow mechanistic study of white matter degeneration, oligodendroglial vulnerability, myelin debris clearance, and cognitive impairment.^21^ These models may be especially suited to testing whether endolysosomal overload in microglia, astrocytes, or oligodendrocyte lineage cells impairs repair and promotes chronic cognitive decline. Because these features do not emerge uniformly across all models, model selection should be hypothesis‐driven rather than convenience‐driven, within an integrated framework linking ischemic injury, NVU trafficking disturbances, and delayed cognitive outcomes (Figure 2).

**FIGURE 2:**
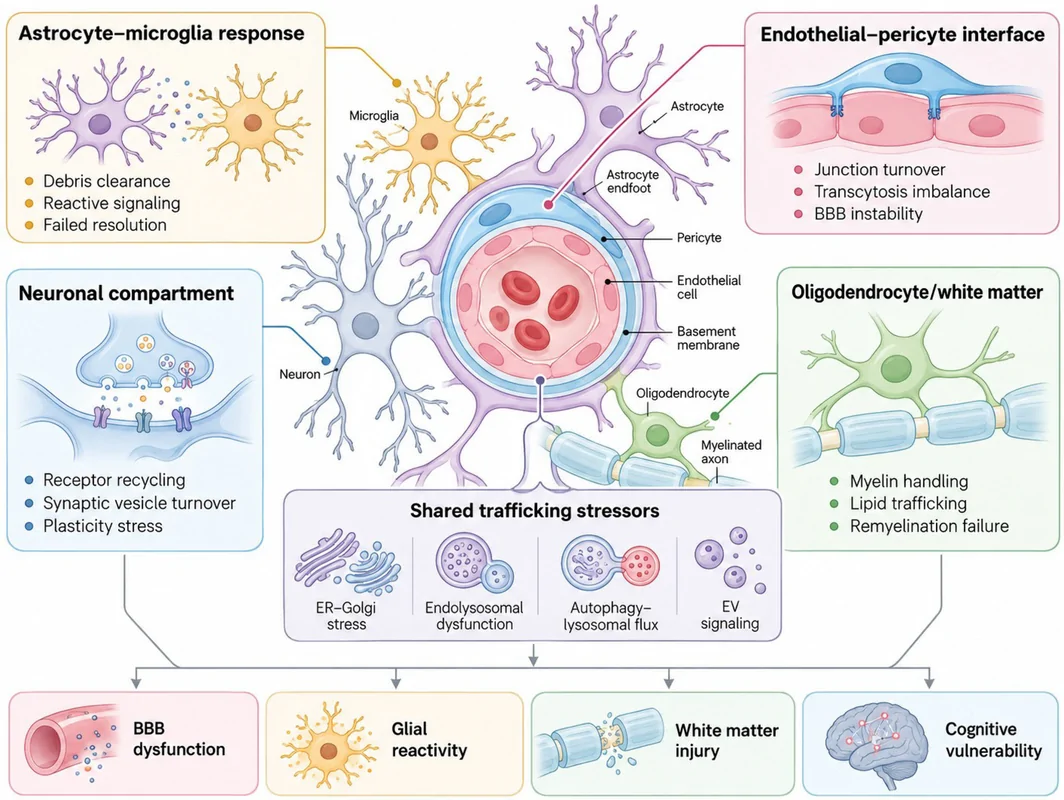
Neurovascular trafficking framework for post‐stroke cognitive impairment.

## 5 CONCEPTUAL FOUNDATION: TRAFFICKING FAILURE AS A TESTABLE ANIMAL‐MODEL MECHANISM

Cerebral ischemia disrupts not only perfusion and excitatory homeostasis but also the intracellular systems that govern membrane trafficking, organelle integrity, protein turnover, and cargo clearance. Within the NVU, these processes are closely linked to BBB maintenance, receptor trafficking, autophagic turnover, synaptic function, inflammatory signaling, and intercellular communication. Disturbance of endosomal and autophagic–lysosomal pathways is increasingly recognized as an important component of ischemic brain injury, providing a mechanistic basis for considering secretory–endolysosomal dysfunction as a potential contributor to post‐ischemic tissue vulnerability.^51^

The functional consequences of trafficking failure are likely to differ across NVU cell populations. In endothelial cells, disruption of endosomal maturation and lysosomal degradation may alter the turnover of adherens and tight‐junction proteins and thereby compromise barrier integrity. This concept is supported by cell‐specific genetic evidence showing that endothelial Rab7a regulates degradation of selected BBB junctional proteins after ischemic stroke.^52^ In astrocytes and microglia, impaired autophagic and lysosomal processing may reduce debris clearance and prolong reactive or inflammatory signaling, whereas neuronal trafficking defects may disrupt receptor recycling, synaptic vesicle turnover, and activity‐dependent plasticity. Oligodendrocyte lineage cells may be particularly sensitive to impaired lipid trafficking and lysosomal processing because myelin maintenance and remyelination require efficient handling of membrane‐rich material.

These observations, however, remain insufficient to establish a causal contribution to PSCI. Temporal association between trafficking abnormalities and cognitive decline may simply reflect the severity of the primary ischemic insult. Causal testing therefore requires targeted perturbation of defined trafficking pathways in specific NVU cell populations, followed by assessment of biologically relevant outcomes. Loss‐of‐function approaches using conditional gene deletion, inducible systems, or CRISPR‐based manipulation can test whether specific regulators of endosomal sorting and lysosomal delivery are necessary for BBB disruption, glial dysfunction, synaptic injury, white matter damage, or delayed cognitive impairment. The Rab7a study provides an important proof of principle that selective manipulation of trafficking machinery can modify a defined post‐ischemic phenotype.^52^

Complementary gain‐of‐function experiments are required to determine whether restoration of lysosomal competence can reverse or attenuate the predicted pathological phenotype. Transcription factor EB (TFEB) is a particularly relevant candidate because it coordinates lysosomal biogenesis and autophagy‐related gene expression. Experimental enhancement of lysosomal Ca^2+^ signaling has been shown to promote TFEB nuclear translocation, improve autophagic–lysosomal function, and reduce neuronal injury after cerebral ischemia.^53^ In animal models of PSCI, cell‐type‐specific TFEB activation or overexpression could therefore be used to test whether improving lysosomal capacity preserves BBB integrity, facilitates debris clearance, protects white matter and synaptic structure, and ultimately improves cognitive outcome. Evidence would be especially informative if these benefits occurred without a major reduction in the initial infarct burden.

EV signaling represents a second experimentally tractable component of this framework. EVs can transfer proteins, lipids, regulatory RNAs, and other bioactive cargo among neurons, glial cells, endothelial cells, and peripheral immune populations. Their biological effects are likely to depend on cell of origin, cargo composition, and the temporal phase of ischemic injury. Neuron‐derived small EVs enriched in miR‐100‐5p have been shown to enhance microglial activation and aggravate neuronal injury after stroke,^54^ while microglia‐derived EVs may exert either detrimental or reparative effects depending on cellular state and cargo.^55^ These findings support the concept that extracellular trafficking may participate actively in the propagation or resolution of post‐ischemic NVU dysfunction.

Demonstrating a causal role for EV signaling requires more than measurement of EV abundance or cargo composition. Experimental strategies should manipulate EV biogenesis, release, uptake, or defined cargo in a cell‐specific manner and determine whether downstream vascular, inflammatory, white matter, and cognitive phenotypes are altered. Rescue or add‐back experiments would provide stronger evidence by testing whether reintroduction of purified EVs or specific cargo restores the phenotype after EV release has been suppressed. Such designs would help distinguish EVs that merely reflect cellular stress from those that actively transmit pathogenic or reparative signals across the NVU.

Temporal control is equally important. Manipulations introduced before or immediately after ischemia are informative for acute injury mechanisms but may not distinguish effects on the primary infarct from mechanisms that contribute specifically to delayed cognitive decline. Inducible interventions initiated after stabilization of the ischemic lesion are therefore particularly valuable. If delayed modulation of Rab‐dependent trafficking, TFEB‐mediated lysosomal function, or EV signaling alters subsequent BBB dysfunction, white matter degeneration, network disconnection, or cognitive decline without substantially changing initial infarct size, this would provide stronger evidence that trafficking dysfunction contributes independently to PSCI. Lesion‐volume matching and, where appropriate, mediation analysis may further help separate effects on initial injury severity from effects on delayed cognitive outcomes.

A rigorous causal framework should therefore integrate longitudinal profiling with cell‐type‐specific loss‐of‐function, gain‐of‐function, rescue, and temporally restricted interventions. These approaches should be combined with behavioral assessment, multimodal imaging, molecular time‐course analysis, EV characterization, and histological evaluation of BBB, synaptic, and white matter integrity. Such experimental designs would move the field beyond descriptive association and allow direct testing of whether secretory–endolysosomal dysfunction is mechanistically involved in the transition from ischemic NVU injury to delayed cognitive vulnerability.

## 6 THE NVU AS A SECRETORY‐ENDOLYSOSOMAL NETWORK IN ANIMAL MODELS

The NVU is a multicellular communication and clearance system. Its secretory arm regulates cytokines, chemokines, growth factors, proteases, metabolic signals, and EVs. Its endolysosomal arm governs receptor recycling, cargo sorting, phagocytosis, autophagy, lysosomal degradation, and proteostasis. In animal models, these processes can be assessed at defined time points and within specific cell populations, allowing direct mechanistic testing that is difficult in human studies.

Endothelial cells are central to NVU failure after ischemia. Beyond tight junctions, they regulate transcytosis, receptor‐mediated transport, inflammatory adhesion, and EV release. Animal models can assess BBB disruption using tracer extravasation, endogenous plasma protein leakage, tight‐junction protein localization, dynamic imaging, and molecular markers of endothelial stress.^7^, ^8^, ^28^, ^29^ These readouts can be linked to lysosomal markers, EV cargo, pericyte coverage, astrocytic endfeet integrity, and later cognition.

Astrocytes provide metabolic, barrier‐supportive, and secretory regulation through their perivascular endfeet. After ischemia, astrocytes may adopt context‐dependent reactive states. Some responses support angiogenesis, synaptogenesis, neurogenesis, and metabolic recovery, whereas others promote inflammation, glial scarring, and restriction of axonal remodeling. EV studies suggest that astrocyte‐derived cargo can be measurable after ischemic stroke, but the prognostic meaning of such cargo for cognition remains incompletely established.^36^, ^37^, ^47^ Animal models can test whether astrocytic EV cargo is repair‐supportive, inflammatory, or merely reflective of tissue injury.

Microglia constitute the immune‐clearance arm of the NVU. Their functions rely on phagocytosis, autophagy, lysosomal degradation, cytokine release, and vesicle‐mediated signaling. Following ischemia or chronic hypoperfusion, microglia are exposed to danger‐associated molecular patterns, damaged synapses, myelin debris, apoptotic cells, and EV cargo. Efficient lysosomal clearance may facilitate debris removal and inflammatory resolution. In contrast, lysosomal dysfunction can impair autophagic flux and promote persistent neuroinflammation.^30^, ^31^, ^32^, ^33^ These processes make microglial lysosomal activity an important candidate mechanism for investigation in models of PSCI and vascular cognitive impairment.

Neurons and oligodendrocyte lineage cells are also integral components of this network. Neurons require tightly regulated exocytosis–endocytosis cycles for neurotransmitter release, receptor recycling, synaptic vesicle turnover, and activity‐dependent plasticity. Oligodendrocytes and their precursor cells depend on lipid handling, myelin protein processing, and lysosomal clearance to maintain and repair myelin. Cognition depends on both synaptic integrity and long‐range white matter connectivity. Therefore, animal models should examine neuronal and oligodendroglial trafficking dysfunction alongside glial and vascular alterations, rather than treating cognitive impairment as a purely neuronal endpoint.

## 7 MECHANISTIC AXES AND ANIMAL‐MODEL READOUTS

Four interconnected trafficking processes provide experimentally tractable mechanistic axes within the ischemic NVU: ER–Golgi secretory stress, endolysosomal dysfunction, autophagy–lysosomal flux, and EV signaling (Figure 3).

**FIGURE 3:**
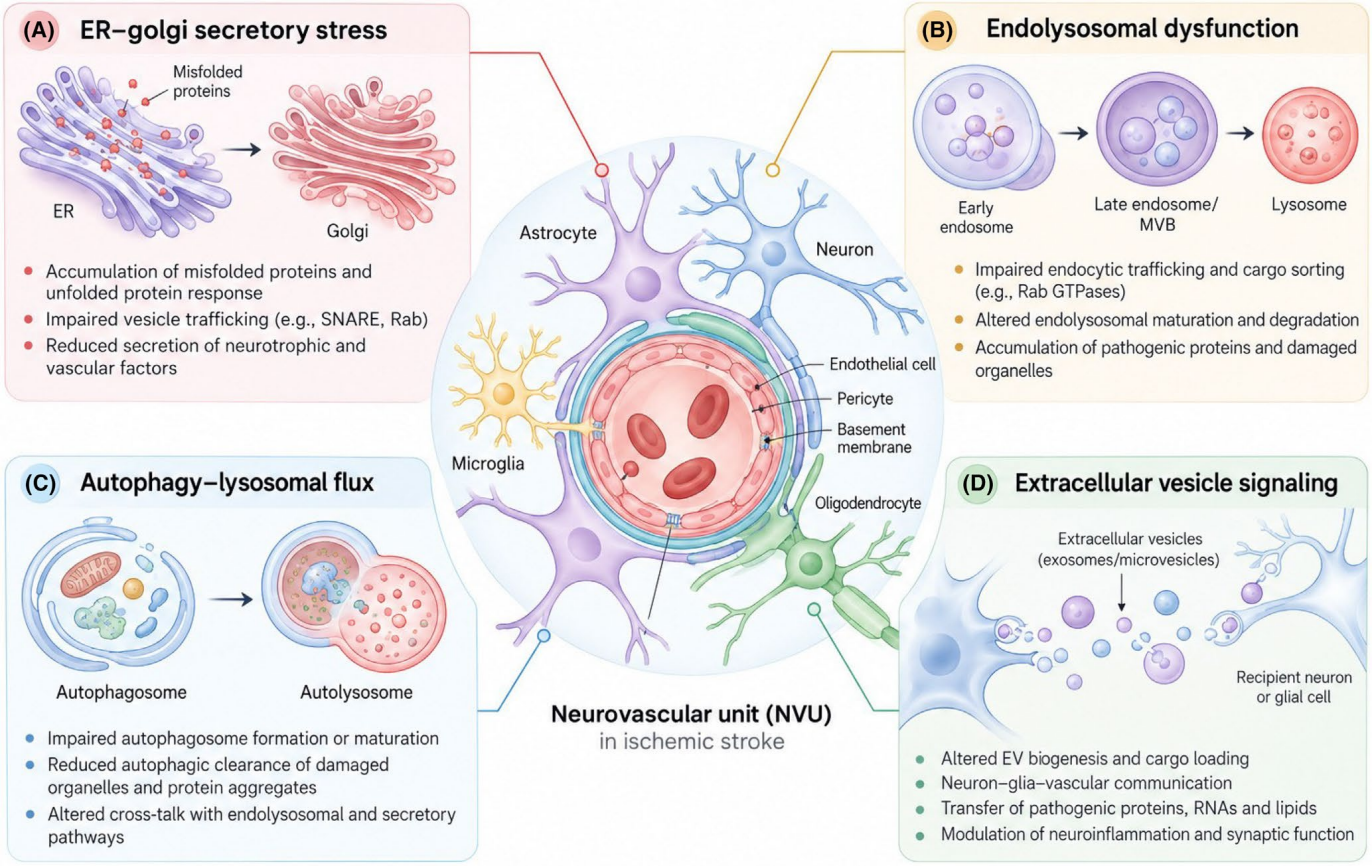
Cellular trafficking mechanisms within the neurovascular unit after ischemic stroke. (A) ER–Golgi secretory stress may impair protein processing and vesicular transport. (B) Endolysosomal dysfunction can disrupt cargo sorting, maturation, and degradation. (C) Impaired autophagy–lysosomal flux may reduce the clearance of damaged organelles and protein aggregates. (D) Altered extracellular vesicle signaling may modify neuron–glia–vascular communication through changes in vesicle biogenesis, cargo loading, release, and uptake. Together, these pathways represent experimentally tractable mechanisms that may contribute to NVU dysfunction and post‐stroke cognitive impairment.

### 7.1 ER‐Golgi stress and secretory pathway disruption

The ER‐Golgi system supports protein folding, post‐translational processing, receptor delivery, cytokine release, trophic signaling, and extracellular matrix remodeling. During ischemia, ATP depletion, calcium dyshomeostasis, oxidative injury, and protein misfolding activate ER stress and the unfolded protein response.^34^, ^35^, ^44^ Golgi stress may add a second layer of disruption by impairing cargo sorting, vesicle formation, glycosylation, and delivery of membrane or secreted proteins.^34^

Animal studies can evaluate this axis by measuring markers such as GRP78/BiP, CHOP, ATF4, ATF6, IRE1, phosphorylated eIF2α, Golgi fragmentation markers, cargo sorting defects, and secreted inflammatory mediators. The most informative designs would relate these markers to BBB leakage, glial activation, synaptic injury, white matter damage, and cognitive performance at later time points. This is crucial because ER‐Golgi stress is well supported as an ischemic injury pathway, but its independent contribution to PSCI remains insufficiently established.

### 7.2 Endolysosomal trafficking, autophagy, and clearance failure

Ischemia produces damaged proteins, oxidized lipids, dysfunctional mitochondria, apoptotic material, synaptic fragments, plasma‐derived proteins, and myelin debris. Under physiological conditions, these materials are sorted through endosomes, recycled when useful, or degraded through lysosomal and autophagy‐dependent pathways. After ischemia, the same cells that must clear injury‐associated cargo are exposed to energy failure, calcium overload, oxidative damage, and inflammatory stimulation, all of which may compromise endosomal maturation, lysosomal acidification, autophagic flux, and proteostasis.^25^, ^26^, ^27^, ^32^, ^33^

Animal studies should move beyond static measurements of LC3, p62/SQSTM1, or the LC3‐II/LC3‐I ratio, because these endpoints alone cannot distinguish increased autophagosome formation from impaired downstream degradation. Dynamic assessment of autophagic flux is therefore required when claiming autophagic dysfunction. Where experimentally appropriate, lysosomal or late‐stage flux blockade using agents such as chloroquine or bafilomycin A1 can be used to assess autophagosome turnover by comparing LC3‐II accumulation in the presence and absence of lysosomal inhibition. These measurements should be interpreted together with complementary readouts, including p62/SQSTM1 turnover, Rab5/Rab7 transition, LAMP1/2, lysosomal pH, cathepsin activity, autophagosome–lysosome fusion, mitophagy markers, and cell‐type‐specific localization. Because pharmacological flux inhibitors may themselves influence ischemic injury and systemic physiology, dose, timing, tissue penetration, and appropriate vehicle controls should be carefully considered in vivo.

### 7.3 EVs as coupling mechanisms

EVs are a point of convergence because they are generated through secretory and endosomal pathways, while their uptake imposes a processing burden on recipient cells. In ischemic stroke, NVU‐derived EVs may carry proteins, lipids, mRNAs, microRNAs, mitochondrial components, inflammatory mediators, complement‐related molecules, and stress‐associated cargo.^36^, ^37^, ^45^, ^46^ Depending on timing, source, and cargo composition, they may support angiogenesis, neurogenesis, oligodendrogenesis, synaptic plasticity, and repair, or propagate BBB‐disruptive and inflammatory signals.

Animal models allow EV source, timing, cargo, and biological activity to be linked to tissue and behavioral endpoints. However, EV studies must be interpreted cautiously because isolation methods, normalization strategies, cell‐of‐origin assignment, and cargo quantification remain technically challenging. Future experiments should standardize EV phenotyping, test biological activity in recipient NVU cells, and determine whether modifying EV cargo changes cognition‐relevant outcomes rather than only reducing infarct volume.

Preclinical EV studies should also be interpreted within the framework of the Minimal Information for Studies of Extracellular Vesicles (MISEV2023) recommendations.^56^ EV characterization should include transparent reporting of sample source, pre‐analytical handling, separation or enrichment procedures, particle and protein characterization, normalization strategy, and functional validation. Particular caution is required when assigning NVU cell of origin. EVs recovered from brain parenchyma may provide closer spatial correspondence to the injured NVU, but tissue dissociation and extracellular matrix disruption can alter vesicle yield and composition. In contrast, circulating EVs are more accessible for longitudinal analysis but represent a heterogeneous mixture derived from neural, vascular, immune, and peripheral tissues. Consequently, putative endothelial‐, astrocyte‐, microglia‐, or neuron‐derived EV populations should not be defined by a single surface marker alone. Orthogonal evidence, including multiple cell‐associated markers, cargo profiles, functional uptake assays, or genetic lineage‐tracing approaches where feasible, is needed to strengthen cell‐of‐origin attribution.

## 8 COGNITIVE PHENOTYPING IN ANIMAL MODELS OF PSCI

Cognitive phenotyping remains one of the most challenging aspects of PSCI research in animal models. Experimental stroke often produces sensorimotor deficits, fatigue, altered exploration, anxiety‐like behavior, visual disturbances, and changes in motivation, all of which can influence performance on cognitive tasks. For this reason, behavioral impairment should not be interpreted as cognitive dysfunction without first considering relevant sensory and motor limitations. Task selection should therefore be guided by the cognitive domain of interest, lesion location, and severity of neurological impairment, with locomotor, sensory, and motivational measures incorporated into the overall interpretation.

A range of behavioral assays is available, including the Morris water maze, Barnes maze, novel object recognition, object location memory, Y‐maze or T‐maze spontaneous alternation, contextual fear conditioning, operant tasks, and touchscreen‐based paradigms. Each has distinct strengths and limitations. In the Morris water maze, for example, prolonged escape latency may reflect impaired swimming ability, visual dysfunction, or altered search strategy rather than a primary deficit in spatial learning. Visible‐platform testing can help identify such confounds, while swimming speed, path length, latency, and search strategy should be examined separately. Similarly, performance in novel object recognition and object location tasks should be interpreted alongside total exploration time and baseline object preference, whereas spontaneous alternation should be considered in relation to total arm entries.

The timing of behavioral assessment is also critical. During the early post‐stroke period, cognitive performance may be heavily influenced by residual neurological impairment. When delayed PSCI is the primary outcome, cognition‐specific testing is therefore most informative after sensorimotor function has stabilized sufficiently to allow valid task engagement. Baseline testing before injury is desirable whenever feasible because it provides a reference for individual change over time. Subacute assessments may capture early impairment and compensatory responses, whereas chronic testing is needed to determine whether cognitive deficits persist or emerge after the primary ischemic injury has stabilized.

Touchscreen‐based paradigms are particularly attractive for translational studies because they allow assessment of cognitive domains such as visual discrimination, paired‐associate learning, reversal learning, attention, working memory, and response inhibition using standardized operant procedures. These tasks can be aligned more closely with human neuropsychological constructs and have been used to demonstrate comparable chronic cognitive deficits in mice and stroke survivors.^15^, ^16^ They may also reduce reliance on complex locomotor output compared with conventional maze‐based tasks, although visual capacity, motivation, and response initiation still require careful control. Recent multicenter work further shows that the detection of post‐stroke cognitive deficits depends strongly on task selection, testing interval, age, lesion severity, stroke model, and methodological standardization.^17^

Within the proposed secretory–endolysosomal NVU framework, longitudinal cognitive phenotyping is especially important because mechanistic interpretation depends on linking cellular dysfunction to the timing and progression of behavioral decline. Cognitive trajectories should therefore be examined together with molecular, vascular, and histological outcomes obtained in parallel cohorts or at predefined terminal time points. Relevant measures include BBB integrity, EV abundance and cargo, autophagic–lysosomal flux, white matter injury, synaptic integrity, and network‐level changes. The most informative designs will distinguish cognition‐specific impairment from residual sensorimotor disability and determine whether trafficking‐related abnormalities predict delayed cognitive decline independently of lesion severity. Evidence for PSCI is therefore strongest when domain‐appropriate cognitive measures converge with careful control of behavioral confounders and biologically relevant NVU endpoints. Recommended cognitive and biological endpoints, together with their interpretive value and major experimental confounders, are summarized in Table 2.

**TABLE 2:** Cognitive and biological endpoints recommended for animal‐model studies of secretory‐endolysosomal dysfunction and PSCI.

Endpoint domain	Recommended measures	Main interpretive value	Common confounds/cautions
Cognitive behavior	Barnes maze, Morris water maze, novel object recognition, object location memory, Y‐maze, touchscreen paired‐associate learning	Detects memory, recognition, spatial learning, attention, and executive‐like deficits	Motor deficits, vision, anxiety, motivation, fatigue, lesion location, baseline variability
BBB/vascular integrity	Tracer extravasation, IgG/fibrinogen leakage, tight‐junction proteins, endothelial activation markers, perfusion imaging	Links endothelial trafficking and barrier failure to cognitive vulnerability	Acute leakage may reflect injury severity rather than chronic mechanism
Secretory pathway stress	GRP78/BiP, CHOP, ATF4/6, IRE1, p‐eIF2α, Golgi morphology, secreted cytokines/proteases	Tests whether ER‐Golgi stress precedes maladaptive communication	Markers are cell‐type nonspecific unless combined with immunophenotyping or sorting
Endolysosomal/autophagy flux	Rab5/Rab7, LC3‐II turnover, p62, LAMP1/2, cathepsins, lysosomal pH, mitophagy markers	Assesses cargo processing and clearance failure	Static marker levels can be misleading without flux assessment
EV biology	Particle number, size, tetraspanins, cell‐origin markers, miRNA/protein cargo, mitochondrial cargo, functional uptake assays	Tests whether EVs transmit stress or repair signals across the NVU	Isolation and normalization methods strongly influence results
White matter and network injury	MBP, PLP, oligodendrocyte lineage markers, myelin debris, axonal markers, DTI‐like imaging, functional connectivity	Connects cellular clearance failure to network disconnection and cognition	White matter injury may be model‐ and strain‐dependent

## 9 PATHOPHYSIOLOGIC OUTPUTS TO BE TESTED IN ANIMAL MODELS

### 9.1 BBB and neurovascular instability

BBB dysfunction is often described as a vascular consequence of ischemia, but it can also be interpreted as an output of disturbed endothelial trafficking and secretion. Endothelial barrier function depends on tight‐junction turnover, vesicular transcytosis, receptor‐mediated transport, cytoskeletal organization, and regulated release of inflammatory or proteolytic mediators.^7^, ^8^, ^28^, ^29^ Animal models can determine whether BBB dysfunction is transient, persistent, or recurrent, and whether its time course relates to lysosomal stress, EV cargo, glial activation, white matter damage, and cognitive decline.

### 9.2 Glial reactivity and failed inflammatory resolution

Post‐ischemic inflammation is not inherently detrimental. Early inflammatory responses participate in debris recognition, phagocytosis, vascular remodeling, and repair. Cognitive risk may arise when inflammatory signaling persists beyond the phase in which it is adaptive. Animal models can test whether incomplete lysosomal processing, defective myelin debris clearance, or persistent EV‐mediated stress signaling maintains microglia and astrocytes in reactive states.^30^, ^31^, ^32^, ^33^

### 9.3 White matter vulnerability and network disconnection

White matter injury provides an anatomical bridge between vascular injury and cognitive decline. Oligodendrocytes and oligodendrocyte precursor cells are vulnerable to oxidative stress, excitotoxicity, inflammatory mediators, and metabolic insufficiency. Effective white matter repair depends on microglial and astrocytic clearance of myelin debris followed by a permissive environment for remyelination. When clearance is incomplete, myelin debris and oxidized lipids may persist and contribute to network disconnection.^4^, ^5^, ^6^, ^18^, ^19^, ^20^, ^21^ Incomplete myelin debris clearance may also alter EV release and cargo composition, potentially enabling inflammatory and stress‐related signals to propagate from injured white matter to other NVU compartments.

Animal models of chronic hypoperfusion, BCAS, and subcortical infarction are especially suited to this output because they reproduce aspects of white matter injury and vascular cognitive impairment. These models can test whether cellular trafficking stress predicts white matter degeneration and whether interventions targeting autophagy‐lysosomal flux improve remyelination, connectivity, and cognition.

### 9.4 Comorbidity‐dependent priming of secretory–endolysosomal vulnerability

Comorbidities may increase post‐stroke cognitive vulnerability not only by worsening ischemic injury, but also by altering the pre‐ischemic state of the NVU. Chronic hypertension and type 2 diabetes are associated with endothelial dysfunction, mitochondrial stress, persistent inflammation, and impaired autophagic–lysosomal homeostasis. These abnormalities may reduce the capacity of NVU cells to maintain intracellular trafficking, organelle quality control, and degradative function when additional ischemic stress occurs.

Hypertensive models provide evidence for such pre‐existing vulnerability. Stroke‐prone spontaneously hypertensive rats exhibit defective autophagy and mitophagy, mitochondrial dysfunction, and NAD^+^ depletion before spontaneous stroke, while pharmacological restoration of autophagy reduces stroke occurrence.^57^ Hypertension also modifies the response to cerebral ischemia, interacting with perfusion deficits to influence tissue outcome^58^ and enhancing inflammatory and neuronal injury after ischemia–reperfusion.^59^ These findings suggest that chronic vascular stress may lower the functional reserve of trafficking and degradative pathways before stroke.

Type 2 diabetes may produce a comparable vulnerability through metabolic mechanisms. The db/db mouse shows chronic hyperglycemia, insulin resistance, vascular dysfunction, and alterations in cortical cell composition and molecular programs associated with cognitive impairment.^60^ In diabetic ischemic models, increased perivascular extracellular NAMPT signaling has been linked to greater neuronal and vascular injury,^61^ while hyperglycemia exacerbates ischemia–reperfusion injury through dysregulated autophagy‐related signaling.^62^ Thus, metabolic stress may amplify post‐ischemic injury by disrupting both intracellular clearance mechanisms and intercellular signaling.

These observations indicate that secretory–endolysosomal dysfunction in comorbid models should be evaluated relative to the pre‐ischemic baseline rather than interpreted solely as a consequence of stroke. Longitudinal studies comparing control and comorbid animals across baseline, acute, subacute, and chronic phases should assess Rab‐dependent trafficking, lysosomal function, autophagic flux, TFEB activity, EV signaling, BBB integrity, mitochondrial function, and inflammatory responses. Such designs are necessary to distinguish pre‐existing deficits from exaggerated injury responses or impaired recovery, and to determine whether reduced secretory–endolysosomal reserve contributes independently to PSCI susceptibility.

## 10 TRANSLATIONAL RIGOR, CAUSAL TESTING, AND WELFARE REFINEMENT RECOMMENDATIONS

Translational rigor, causal inference, and welfare refinement should be integrated from the outset when designing animal studies of PSCI and secretory–endolysosomal dysfunction. ARRIVE 2.0, SYRCLE, and STAIR‐related recommendations emphasize randomization, allocation concealment, blinded outcome assessment, sample‐size justification, predefined inclusion and exclusion criteria, mortality reporting, and the use of clinically relevant outcome measures.^38^, ^39^, ^40^, ^41^, ^42^, ^43^ These safeguards are particularly important in experimental stroke, where variability in infarct size, surgical mortality, body temperature, hydration, weight loss, motor impairment, and selective attrition can substantially influence both molecular and behavioral outcomes.

A rigorous PSCI study should therefore combine baseline cognitive assessment with an injury model selected to match the biological question, predefined analytical criteria, and appropriate control of major sources of bias. Acute, subacute, and chronic time points are needed to distinguish early injury mechanisms from processes that emerge during delayed cognitive decline. Both sexes should be included when biologically and statistically justified, and aged or comorbid animals should be incorporated when translational relevance is a primary objective. Molecular, imaging, histological, and behavioral endpoints may require parallel or adequately powered cohorts, particularly when repeated tissue sampling is not feasible. Exclusion criteria related to technical failure, lesion characteristics, mortality, or welfare endpoints should be specified prospectively rather than after inspection of the data.

Longitudinal association alone, however, is insufficient to establish that secretory–endolysosomal dysfunction contributes causally to PSCI. Mechanistic studies should incorporate cell‐type‐specific loss‐of‐function and gain‐of‐function approaches. Rab‐family GTPases are particularly informative targets because they regulate distinct stages of endosomal sorting, recycling, maturation, and lysosomal delivery. Endothelial‐specific deletion of Rab7a has already shown that selective disruption of a trafficking regulator can alter post‐ischemic BBB pathology.^52^ Complementary gain‐of‐function studies should test whether restoration of lysosomal competence can reverse the predicted phenotype. TFEB is especially relevant in this context because of its central role in lysosomal biogenesis and autophagy‐related gene expression; enhancement of TFEB‐dependent lysosomal function has been associated with improved autophagic–lysosomal processing and reduced ischemic injury.^53^

EV signaling should be examined using the same causal framework. Measurements of EV abundance or cargo can identify candidate pathways, but they remain associative unless EV production, release, uptake, or cargo transfer is experimentally manipulated. Cell‐specific inhibition of EV‐release machinery, followed by rescue or add‐back of purified EVs or defined cargo, would provide stronger evidence that EV signaling contributes directly to downstream vascular, inflammatory, white matter, or cognitive phenotypes. Such experiments are particularly valuable when they demonstrate pathway necessity or reversibility rather than simply changes in EV profiles.

Temporal control is equally important for causal interpretation. Manipulations initiated before or immediately after ischemia are informative for acute injury mechanisms but may not distinguish effects on the primary infarct from those on delayed cognitive decline. Inducible interventions introduced after stabilization of the ischemic lesion are therefore more informative for testing PSCI‐specific mechanisms. If delayed modulation of Rab‐dependent trafficking, TFEB‐mediated lysosomal function, or EV signaling alters subsequent BBB dysfunction, white matter degeneration, network disconnection, or cognition without materially affecting initial infarct size, the evidence for an independent contribution to PSCI would be substantially strengthened. Rescue, reversal, and pathway‐interaction experiments can further clarify mechanistic ordering and distinguish pathway necessity from sufficiency.

The central question is therefore not whether trafficking abnormalities accompany cognitive decline, but whether experimentally modifying these abnormalities changes cognition‐relevant outcomes. A mechanism would gain stronger support if restoration of lysosomal function reduced persistent glial activation, improved myelin debris clearance, preserved white matter integrity, and improved cognition independently of major effects on infarct size. Conversely, if trafficking markers simply scale with lesion severity and lose predictive value after accounting for infarct burden and motor impairment, the proposed framework should be narrowed accordingly.

Experimental rigor should also be accompanied by explicit welfare refinement. Stroke models may produce dehydration, hypothermia, weight loss, feeding difficulties, pain, impaired mobility, and increased mortality, all of which can compromise welfare while introducing biological variability. Perioperative monitoring, hydration and nutritional support, temperature control, predefined humane endpoints, and analgesic or supportive strategies compatible with the planned outcomes should therefore be incorporated prospectively. Welfare‐related variables should also be reported transparently because differences in postoperative care may influence inflammation, metabolism, recovery, and cognitive performance.

Ultimately, the quality of an animal model of PSCI should not be judged solely by its ability to produce an ischemic lesion or a behavioral deficit. A robust model should demonstrate face validity for the targeted PSCI phenotype, construct validity for the proposed NVU mechanism, and predictive validity for intervention response. For mechanistic claims, these forms of validity should be complemented by appropriately controlled loss‐of‐function, gain‐of‐function, rescue, and temporally restricted experiments. Integrating causal testing with rigorous study design and welfare refinement will strengthen both the biological interpretation and translational relevance of secretory–endolysosomal mechanisms in PSCI.

## 11 TRANSLATIONAL IMPLICATIONS AND LIMITATIONS

The animal‐model‐centered framework has translational value because it identifies testable biomarkers and therapeutic targets while avoiding premature clinical claims. EV cargo, lysosomal stress markers, BBB measures, white matter imaging, and cognitive testing should be considered candidate research tools rather than validated clinical biomarkers. Their utility depends on longitudinal validation, standardized methods, and evidence that they add predictive value beyond lesion volume, age, vascular risk, white matter burden, and baseline cognitive reserve.^1^, ^2^, ^4^, ^5^, ^6^, ^28^, ^29^, ^36^, ^37^, ^47^, ^48^, ^49^

Several limitations must be acknowledged. First, PSCI in humans is heterogeneous and often develops in the context of aging, hypertension, diabetes, atrial fibrillation, small vessel disease, depression, sleep disturbance, and neurodegenerative co‐pathology. Young healthy rodents do not capture this complexity. Second, cognitive tasks in animals measure specific behavioral constructs that only partially correspond to human cognitive domains. Third, molecular readouts of autophagy, lysosomal stress, EV cargo, and ER‐Golgi dysfunction are sensitive to timing, cell type, tissue processing, and normalization strategies. Fourth, reducing infarct volume does not necessarily demonstrate protection against delayed cognitive decline. Fifth, a model may have strong face validity for focal ischemia but limited construct or predictive validity for delayed PSCI. Finally, EV‐based or lysosomal‐modulating therapies require careful control of source, dose, timing, biodistribution, immunogenicity, safety, welfare burden, and functional endpoints before clinical translation.^34^, ^35^, ^36^, ^37^, ^47^, ^48^, ^49^

## 12 TESTABLE PREDICTIONS AND FUTURE DIRECTIONS

The revised framework yields several experimentally testable predictions. First, early post‐ischemic markers of endolysosomal stress should predict later BBB disruption, white matter progression, or cognitive impairment if the pathway is clinically relevant. Second, EV cargo should show temporal and cell‐of‐origin specificity across acute injury, subacute repair, and chronic maladaptation. Third, interventions that normalize lysosomal flux or EV cargo in experimental NVU systems should improve barrier integrity, reduce unresolved glial activation, enhance debris clearance, preserve white matter integrity, or improve cognition‐relevant behavioral outcomes. Fourth, animal models incorporating aging, hypertension, diabetes, or chronic hypoperfusion should show stronger and more clinically relevant secretory‐endolysosomal vulnerability than young healthy focal‐stroke models alone.

Future studies should also integrate in vivo models with human‐relevant platforms such as multicellular co‐cultures, induced pluripotent stem cell‐derived NVU systems, organoid‐based vascular models, and NVU‐on‐chip systems.^63^ These platforms cannot replace animals, but they can refine cell‐type‐specific hypotheses, reduce animal use, improve mechanistic precision before in vivo testing, and strengthen alignment with the 3Rs: replacement, reduction, and refinement.

## 13 CONCLUSION

Secretory‐endolysosomal dysfunction in the ischemic NVU offers a biologically plausible, hypothesis‐generating framework for understanding how acute cerebral ischemia and chronic vascular stress could contribute to delayed cognitive vulnerability. The present review reframes this framework around animal models and emphasizes that progress in PSCI research depends on the integration of model validity, translational rigor, and welfare refinement. Rather than asserting a proven causal pathway, it argues that animal models provide the experimental leverage needed to test whether impaired secretion, trafficking, EV‐mediated signaling, autophagy‐lysosomal flux, BBB repair, glial clearance, and white matter maintenance contribute independently to PSCI. The most valuable future studies will combine carefully selected animal models, longitudinal cognitive phenotyping, cell‐type‐specific molecular analysis, EV characterization, multimodal imaging, intervention‐based causal validation, transparent reporting, and refinement strategies that improve both scientific reliability and animal welfare.

## AUTHOR CONTRIBUTIONS

**Pratchaya Kaewkaen:** Conceptualization; software; project administration; visualization; validation; writing – original draft; investigation; writing – review and editing; methodology; formal analysis; data curation; supervision; resources.

## FUNDING INFORMATION

No funding was received for this work.

## CONFLICT OF INTEREST STATEMENT

The author declares no conflict of interest.

## ETHICS STATEMENT

This review did not involve new animal experiments or human participants.

## DECLARATION OF GENERATIVE AI IN SCIENTIFIC WRITING

The scientific writing, conceptual framework, and original figure designs were developed by the author. ChatGPT (GPT‐5.6 Sol, OpenAI) was used solely as an assistive tool to refine graphical organization, layout, labeling, and visual clarity. The tool was not used to generate the scientific concepts, interpretations, or conclusions. All AI‐assisted outputs were critically reviewed, revised, and finalized by the author, who takes full responsibility for the final manuscript and figures.

## Data Availability

The data that support the findings of this study are available from the corresponding author upon reasonable request.
